# Supplementary material for: Are carbon emissions trading and green financial instruments synergistic? -Comprehensive quantitative research based on content analysis
Source: PLoS One. 2024 Mar 7;19(3):e0298601. doi: 10.1371/journal.pone.0298601 (PMC10919601; doi:10.1371/journal.pone.0298601)
Supplement: S1 Data — (ZIP) [file pone.0298601.s001.zip › Data/地级市Z_F工作报告.zip!/║■▒▒/╧╠─■╩╨/└┤╫╘╣1⁄2╓┌║┼í╛┬φ┐╦╩2╛▌═°í┐_2015.docx]

各位代表：

现在，我代表市人民政府，向大会作政府工作报告，请予审议，并请市政协委员和其他列席人员提出意见。

一、2014年工作回顾

过去一年，在省委、省政府和市委的坚强领导下，市政府团结带领全市人民，认真贯彻落实党中央、国务院重大决策部署和省委、省政府重要工作部署，从容应对挑战，奋力攻坚克难，适应经济发展新常态取得良好开端，全面深化改革实现良好开局，市四届人大四次会议确定的目标任务较好完成。

——综合实力稳步攀升。初步测算，全市生产总值1000亿元，增长9.5%；全社会固定资产投资1148.75亿元，增长20.5%；社会消费品零售总额361.77亿元，增长12.8%；一般公共预算收入70.76亿元，增长20.5%，其中市本级超过20亿元。

——项目建设提速见效。全市固定资产投资额2000万元以上新签约工业项目314个、新开工工业项目278个、新投产工业项目245个，全面完成目标任务。中航国际、中电华基、中天引控、新兴际华等央企合作项目成功落户，南玻节能、天化麻业、咸安洪盛模具、通山水晶文化工业园加速扩张，通城玉立樊牌、崇阳兴民钢圈建成投产，香泉映月、嘉鱼金盛兰冶金、赤壁万吨特种新材料再创“咸宁速度”。建成总投资2.27万亿元项目储备库，为全市下一个五年发展打下了坚实基础。

——生态环境增创优势。在全国率先编制绿色崛起发展规划，成功申报国家生态保护和建设示范区。节能减排完成省定目标。全市森林覆盖率增长0.23个百分点。市区环境空气质量优良天数达到324天，居全省第二位。富水湖、向阳湖成功创建国家湿地公园，崇阳桂花林场成功创建国家森林公园。引进高端资源打造绿色发展新模式，全国首个林业生态产业（文化）产权交易中心落户咸宁。

——人民生活持续改善。公共财政支出用于民生的比重达到69.1%。城乡居民人均可支配收入分别增长10%和12%，高于GDP增速。继续上调企业退休、退职人员基本养老金，启动农村特困居民大病医疗救助，机关和事业单位规范津补贴稳步实施。各项强农惠农政策全面落实。物价水平总体平稳。平安、法治建设取得明显成效。

一年来，我们坚持以改革创新统揽全局，主动适应经济发展新常态，主要做了以下工作：

（一）着力推动产业转型。坚持做大总量和优化结构并重，推动发展方式转变取得实质性成效。全市三次产业结构调整为17.5：49.9：32.6。大力推进工业崛起，重点实施“一工程三计划”。规模以上工业增加值480亿元，增长10.5%；新增规模以上工业企业119家、净增81家，总数861家；咸安现代森工新进入全省重点成长型产业集群。高新技术产业增加值64亿元，占GDP比重提高到6.4%；新认定国家高新技术企业26家、总数61家，赤壁经济开发区晋升省级高新区。企业专利申请量成倍增长，增幅居全省第一位。新增中国驰名商标5件，总数13件、居全省第四位。加快发展现代农业，出台竹、茶、油茶产业发展一系列措施。粮食生产实现“十一连增”。市级以上农业产业化龙头企业188家、新增21家。成功申报国家楠竹生产农业标准化示范区，巨宁森工、黄袍山公司获批国家林业重点龙头企业，赤壁获颁国家有机产品认证示范创建区及中国名茶之乡，羊楼洞砖茶文化成为全省首个“中国重要农业文化遗产”，嘉鱼鱼圆、通山乌骨山羊、咸安南川蜜桔、崇阳野桂花蜜等获国家地理标志产品认证。加快发展现代服务业。接待游客3365万人次，旅游收入172亿元，分别增长26.96%、31.87%。赤壁获批第二批全国旅游标准化示范城市。大润发华中物流基地、通城综合物流园、崇阳天城物流园、赤壁“绿购网”建成运营，天成财富中心、泉都物流中心、咸安商贸物流区建设顺利推进，通山被列为全国电子商务进农村试点县。房地产业持续健康发展。市城区新建商品房成交1.46万套、150.48万平方米，成交额60.11亿元，分别增长19.81%、9.11%、25.2%。

（二）着力激发市场活力。坚持把改革创新贯穿于经济社会发展各领域各环节，推出“1+N”改革行动，23个重大专项改革、100项重点改革事项稳步推进。围绕 “三张清单”，大力简政放权，推动政务服务中心“二次创新”，共取消、暂停、下放行政审批和管理事项731项，减少比率55.2%；市本级行政许可事项下降到87项。出台工商登记“先照后证”等16条新规，优化投资项目审批。全市各类市场主体18.3万户，增长17.9%；新登记企业6213户，增长36%。扎实推进市域金融工程，组建中小企业金融服务中心，设立红土成长基金、高投基金、企业应急循环基金，推出“政银保集合贷”等金融创新产品，启动实施“小钢炮”计划和“无微不至”计划。金融机构贷款余额突破500亿元，贷款增幅居全省第一位，贷存比超过60%，连续2年获评全省金融信用市州第一名。推进香城创业计划，设立大学生创业就业促进中心，25个创业孵化基地、150套创业公寓为创业者插上腾飞的翅膀。完成新一轮市区体制调整，建立健全全口径预算体系，投融资平台加快转型，政府性债务风险总体可控，住房公积金管理良性发展。土地改革新秩序基本建立，争取部、省土地整治面积25.1万亩。国有林场、农村水利、公立医院、事业单位分类改革等稳步推进。坚持以开放促改革、促发展，与武汉的经济联系更加紧密，与长江中游城市群的合作不断深化，国际友城交往更加频繁。实际利用外资5880万美元；出口3.97亿美元，增长36.4%，增幅居全省第二位。

（三）着力统筹城乡发展。在全省率先编制完成《咸宁全域新型城镇化总体规划》，构建了“12255”全域空间格局。围绕打造“香城泉都”，大力实施城市提质工程，咸宁大道东段、十六潭路东段、贺温路综合改造、西外环线、城铁东站和南站站前广场、“锦绣香城”古街、金桂路西段及金桂西桥等工程相继竣工。成功举办第六届国际温泉文化旅游节，创建国家卫生城市工作喜结硕果，城市精细化管理和数字化城管取得进展。推进市域交通枢纽建设，通界高速竣工通车，咸宁（嘉鱼）长江大桥开工在即，武深高速、武汉城市圈环线高速、咸宁通江大道（咸潘一级公路）、武咸快速通道、幕阜山生态旅游公路等项目建设加快。推动城市空间向“四城四区”拓展，梓山湖生态科技新城、旅游新城、咸嘉临港新城面貌一新，大洲湖湿地生态保护区建设启动。抓好重点镇、特色镇建设，11个镇列入全国重点镇名单，嘉鱼潘家湾率先完成“四化同步”试点全域规划编制，15个省级重点中心镇、特色镇试点示范进展良好。探索建设美丽乡村，完善通村道路、供水、绿化、垃圾处理等基础设施。“三万”活动扎实开展。农村网格化管理全覆盖。7个村庄进入第三批“中国传统村落”名录。完成造林32.88万亩。

（四）着力兴办民生实事。把改善民生放在更加突出位置，筑牢织密民生保障安全网。加强就业和社会保障工作，城镇登记失业率控制在2.57%以内，五项社会保险参保165.4万人次，综合参保率98%。城乡低保标准分别提高7.5%和29.3%，惠及城乡低保对象12.5万户、19.79万人。加大幕阜山片区扶贫攻坚力度，储备项目802个，全市累计减贫6万人。优先发展教育事业，咸宁实验外国语学校建成开学，鄂南高中债务妥善化解，嘉鱼、崇阳、咸安顺利通过省级以上义务教育均衡发展评估验收。深化医药卫生体制改革，新型农村合作医疗参合率100%，“百镇千村”示范卫生机构创建工作扎实推进。建成120急救体系，市区三级急救站启动运行。实施“单独二孩”政策，严格控制政策外生育。加强公共文化服务标准化建设，“一五一十”工程叫响全国，“两馆一站”累计服务群众80万人次。普法依法治理深入推进。村级换届选举全面完成。刘汉刘维黑社会性质组织犯罪在咸宁得到依法处理，承办工作广获好评。食品药品安全监管、信访、安全生产工作进一步加强，社会大局安全稳定。国防动员、国家安全、人民防空工作扎实推进，民族、宗教、外事、侨务、对台、老区、统计、保密、档案、方志、气象、地震、科普等工作全面发展，妇女、儿童、青少年、老龄、残疾人、慈善等事业取得新进步，驻外机构、对口支援、社会科学、政策咨询、机关事务管理等工作，也都取得新的成绩。

2014年十件民生实事全面完成，兑现了向全市人民作出的庄严承诺！

（五）着力转变政府作风。扎实开展第二批党的群众路线教育实践活动，着力整改“四风”突出问题，切实解决关系群众切身利益和联系服务群众“最后一公里”问题。改进调查研究，清理文山会海，政府性会议减少16.2%，文件精简11.5%，市级“三公”经费压缩31.9%。及时向人大报告工作、向政协通报情况，办理人大代表建议49件、政协委员提案120件。加强法治建设，推行网上执法，全面推进政务公开，市本级受理依法申请公开事项71件。深入开展电视问政、治庸问责、行风评议等活动，转变作风优化环境集中整治活动常态化。落实廉政建设责任制，加强惩治和预防腐败体系建设，纠正违纪行为，查处贪腐分子，促进了政府廉政建设。

过去一年，我们付出了艰辛努力，经受住了严峻考验。成绩的取得，得益于市委的坚强领导，得益于市人大、市政协的监督、支持，得益于社会各界、全市人民的共同努力。在此，我代表市人民政府，向全市广大干部群众、离退休老同志，各民主党派、工商联、无党派人士、各人民团体，驻咸部队、武警消防官兵、公安司法干警，以及所有参与、关心和支持咸宁发展的同志们、朋友们，表示衷心的感谢并致以崇高的敬意！

在经济社会发展和政府工作中，我们清醒认识到还存在一些问题和不足，主要是：经济规模不大，产业特色不鲜明，企业自主创新能力不强，市场机制作用发挥不充分；城乡区域发展差距较大，社会事业发展欠账较多，农民增收特别是贫困地区脱贫致富任务艰巨；发展环境和生态环境还存在不少问题；政府效能有待提高，有些公务人员对经济发展、作风建设新常态还不适应等等。对这些问题，我们将加大改革创新力度，采取综合措施切实解决。

二、2015年工作安排

今年是全面深化改革的关键之年，是全面推进依法治国的开局之年，是全面完成“十二五”规划的收官之年，也是全面谋划“十三五”规划的奠基之年。我们发展的外部环境和条件正在发生深刻变化，但处于大有可为的黄金发展期没有改变。从战略层面看，国家强力推进“一带一路”和长江经济带战略，明确支持长江中游城市群和武汉城市圈建设，我市在区域发展格局中的战略地位更加凸显。从政策环境看，国家继续实施积极的财政政策和稳健的货币政策，更加重视向中西部倾斜，特别是随着一系列改革措施的落地，将进一步释放改革红利，更好地促进经济社会持续健康发展。从城乡区域看，我市城乡区域发展不平衡，通过推动基本公共服务均等化拉动经济增长的空间和潜力很大。从社会需求看，信息消费、健康养老、文化娱乐、体育健身等消费热点持续升温，蕴含着巨大的增长空间。从产业升级看，新一轮科技革命和产业变革正在孕育兴起，为我市加快产业升级提供了宝贵机会窗口。从自身发展看，我市总体发展不够，但历届市委、市政府克难奋进，打下了良好的发展基础，凝聚了强大的发展气场，有利于我们在经济发展新常态下实现弯道超越。同时，由于后发而保存良好的生态优势凸显，有利于我们吸引集聚人才、资本、技术等核心要素。只要我们发挥新优势，抢抓新机遇，适应新常态，实现新作为，就一定能够创造出一个大有作为的经济社会发展新局面。

今年政府工作的总体要求是：全面贯彻落实党的十八大、十八届三中四中全会和习近平总书记系列重要讲话精神，按照中央、全省经济工作会议和市委四届四次五次全会的部署，坚持“效速兼取、去稳竞进”工作总基调，牢牢把握市场、绿色、民生“三维”纲要，主动适应经济发展新常态，把转方式调结构放在更加重要位置，狠抓改革攻坚，推进法治建设，突出创新驱动，加强民生保障，全面完成“十二五”规划任务，在推进省级战略咸宁实施中加快实现绿色崛起。

全市经济社会发展主要预期目标是：生产总值增长10%左右；规模以上工业增加值增长12%；固定资产投资增长20%；社会消费品零售总额增长13%；一般公共预算收入增长14%；出口总额增长12%；城镇常住居民人均可支配收入增长10%；农村常住居民人均可支配收入增长10%以上；居民消费价格涨幅控制在3.5%以内；城镇登记失业率控制在4.6%以内；人口自然增长率控制在6.5‰以内；全面完成省定节能减排任务。

实现上述目标，必须全面落实党中央、国务院和省委、省政府以及市委的各项决策部署，以发展的硬道理凝聚思想共识，以各项工作的新作为引领新常态。对咸宁来说，最重要的是做大总量，在加快发展中转方式、调结构。为此，在工作导向上，不能满足于四平八稳，更不能安于现状，要鼓足劲、加把油、拼一拼、跳一跳，努力实现量有突破、质有提升，确保经济增速高于全省平均水平，确保绿色增长走在全省前列。

今年，重点抓好以下七个方面的工作：

（一）保持经济平稳较快发展

积极扩大投资。大力开展“项目建设竞进年”活动，推动形成千方百计抓项目、扩投资的强大气场。抢抓国家推进重点领域改革、长江经济带建设、军民融合发展等机遇，积极谋划一批重大项目，力争中央、省投资项目在全市占据更大份额。突出抓好工业投资，引导企业加大设备更新和技术改造力度，全力推进合加环保等81个省级重大专项快开工、快投产、快见效。认真谋划推进咸宁临江产业园、幕阜山清洁能源基地、光谷南产业基地等项目。围绕打造综合交通枢纽，进一步做好岳咸九铁路、仙崇高速、咸九高速、咸鄂高速等项目前期工作，加快推进武深高速、武汉城市圈环线高速、咸宁通江大道、武咸快速通道、咸崇旅游公路、幕阜山生态旅游公路、咸宁港综合码头等项目建设，确保咸宁（嘉鱼）长江大桥开工建设，力促赤壁长江大桥尽快开工建设。抓好农村电网改造升级、城乡垃圾一体化治理工程建设。发挥政府资金引导作用，鼓励社会资本参与基础设施建设，打造政社合作PPP模式。结合“十三五”规划研究，做深、做实重大项目储备库。

进一步拉动区域消费。坚持城市和农村并举、扩大传统消费和培育新的消费热点相结合，千方百计激活和扩大消费需求，使大众消费“汇小溪成大河”。抓好中心城区商业网点规划编修和县级商业网点规划，优化提升商圈布局。以国家实施养老健康家政、信息、旅游休闲、住房、绿色、教育文化体育等6大领域消费工程为契机，创新消费供给，释放消费潜力，满足多层次、多样化需求。积极落实促进消费各项政策，鼓励开展展销会、购物节、美食节等促销活动，扩大节日消费。深入推进“万村千乡市场工程”信息化建设，加快新世界、大润发等市场建设，推动农产品进超市、入电商。进一步抓好“菜篮子”工程和放心粮油市场建设。加强食品药品及重要消费品市场监管，保障消费安全，让城乡居民愿意消费、敢于消费、放心消费。

提高经济运行质量。加强经济运行监测调度，确保经济平稳较快发展。做大做强咸宁经济开发区，力争工业固定资产投资100亿元以上，有150个以上项目签约、开工、投产。鼓励县（市、区）争先进位，打造县域经济升级版，全力支持赤壁挺进第一方阵。坚持“六个一”工作机制，创新拉练检查方式。强化电、气、运等要素保障，助力企业发展。加强税源管理，推进依法治税，坚持均衡入库。抓好农资、食品等市场供应，保持物价总水平稳定。

（二）促进产业结构优化升级

持续推进工业崛起。围绕实施“一工程三计划”，增强现代装备制造、电子信息、生物医药、新能源、互联网与电商等战略性新兴产业的支撑作用，推动食品饮料、纺织服装、机电制造等传统产业和陶瓷、钒加工、冶金建材等资源型产业转型升级。全力支持华中绿色印刷包装物流产业园、广东工业园中部国际企业城、津晶城、立视幻影科技城、中航产业新城等产业基地建设，促进产业集聚式发展。加强产业链垂直整合，支持和推动咸安环保产业园、嘉鱼长江缆索产业园、赤壁应急装备产业园、通城高新技术工业园、崇阳生物医药产业园、通山水晶文化工业园等专业园区加快发展，提升园区发展水平。加快推进立邦涂料、福人科技、华琪生物、金盛兰冶金、三赢兴电子二期、香溢城酒业、金欧米门业等项目建设，确保奥瑞金制罐、杰士邦、谷都生物、华博阳光、嘉麟杰纺织、金宝丽薄膜、平安电工四期、帛典家纺等项目建成投产。加强中小企业服务体系建设，发挥企业联合会作用。重点扶持100家骨干企业，力争1—2个产业跨入省级重点成长型产业集群行列，新增100家以上规模以上工业企业。

加快发展现代农业。大力发展高效生态型、品牌特色型、休闲观光型农业，千方百计增加农民收入，建设生态农业强市。实施“粮安工程”，稳定粮食生产。以“三大百亿产业”为核心，推动“十万千”农业产业化基地建设，加快推进咸安竹循环经济产业园、赤壁砖茶产业园、黄袍山国家油茶产业发展示范园建设。围绕延长农产品产业链、价值链，实施农产品加工业发展提质工程，推动农产品加工业提档升级，打造农产品加工品牌。完善农企联结机制，大力扶持发展农民专业合作社、专业大户、家庭农场等农业经营主体。推进农作物品种改良和农业科技创新，开展农业标准化建设，提高农业机械化水平。

拓展提升现代服务业。围绕建设旅游经济强市，突破性发展旅游业，争创国家旅游综合改革试点市。加快香泉映月、百盟欢乐谷、际华目的地、室内剧《香城恋》、风情赤壁等重点旅游项目建设，开设城区“一日游”、“二日游”等精品旅游线路，推进赤壁古战场、九宫山风景区创建国家5A级景区，打造一批旅游名镇、名村、名街。积极培育文化游、乡村游、旅游电商等新兴旅游业态，发展“月光经济”，推进旅游产业跨界融合。实现全年接待游客4000万人次以上，旅游经济收入210亿元以上。发展大健康产业，实施“养在咸宁”健康产业培育工程，打造华中健康养生谷。以水公空铁多式联运一体、物流产业新城一体、网上网下市场一体等“三个一体”为模式，以航空物流、港口物流和铁路物流为方向，重点构建“一核二港三中心”，打造华中地区物流枢纽。集中连片推进农产品流通体系建设，大力发展冷链物流、专业市场，继续抓好咸安现代服务业改革试点。推进互联网与电商产业基地建设，推动阿里巴巴、天助网、本地宝等电商企业以及文化创意、设计、会展等相关产业落户我市。大力发展工业地产，稳步推进商业地产、住宅地产开发。

（三）更大力度深化改革开放

全面对接“一带一路”和长江经济带。抢抓国家战略机遇，加强与“一带一路”的对接融合，重点推进基础设施互联互通，同步推进商贸物流服务业发展。支持赤壁建设万里茶道休闲度假区，办好2015年国际茶业大会和中国青（米）砖茶交易会，打造“赤壁青砖茶”公共品牌。借力长江经济带，深化长江中游城市群合作，加快对接大武汉、服务大武汉，打造卫星城。发挥港口、岸线资源优势，加快武汉新港咸宁港区建设，建设咸嘉新城飞地经济区。推动潘家湾新港（物流）工业园创建省级开发区。

推进重点领域和关键环节改革。发挥经济体制改革的牵引作用，推动各方面改革相互促进，使改革举措有效转化为发展动力。进一步简政放权，再取消和下放一批行政审批事项，在咸宁经济开发区开展“一口式”承诺办理制试点。着力打通政策出台实施的“最先一公里”和政策落地的“最后一公里”，有效减少中间环节。按照新《预算法》健全政府预算体系，力推预算绩效管理。规范政府性债务管理，推动投融资平台转型升级。加快党政机关所办企业、中介机构脱钩改制进程，提高国有资产配置效率。改进住房公积金提取、使用、监管机制。抓好工矿废弃地复垦利用和城乡建设用地增减挂钩、低丘缓坡土地综合开发利用试点，加大高产农田整治力度。稳步推进不动产统一登记制度改革。加快推进土地承包经营权确权登记颁证和农村集体土地“三权分置”改革，加强农村产权交易平台建设。深化教育文化、医药卫生、社会保障、收入分配等公共服务领域改革。全面启动机关事业单位工作人员养老保险制度改革。加快户籍制度改革。支持推动司法体制改革。推进公务用车改革。完成市县政府机构改革，加快推进事业单位分类改革。

提高对外开放水平。积极承接沿海地区制造业和特大城市劳动密集型服务业拓展。进一步加强与中航、中电、恒天等央企的对接合作，共建央企合作产业基地。创新招商方式，优化招商环境，提高招商质效，力争在引进世界500强、国内500强、上市公司和行业龙头企业上取得突破。加强出口基地建设，提高地产品出口比重，打造出口优势产业集群。加快推进海关、商检、口岸“三位一体”通关建设，探索设立保税物流园。加快实施“走出去”战略，鼓励企业开展对外投资合作。加强国际经贸文化交流，积极引进海外高层次人才，借助国际友城等资源，拓展国际交往渠道，提升城市开放程度和国际化水平。

（四）推动大众创业、万众创新、全民创富

实施创新驱动发展战略。一切文明成果，都要大胆吸纳；一切新生事物，都要积极对待；一切创新实践，都要热情鼓励。大力开展“科技创新年”活动，争创全国科技进步先进市。以创建国家级高新区为目标，重点构建“1+5+X”高新产业园区协同发展体系，整合长江工业园、咸安、赤壁、嘉鱼、通城等五个园区，打造若干个高新技术产业基地、科技孵化器、工程技术中心和产学研研究院，推进规划协同、产业协同、技术平台协同、机制体制协同。进一步加大对自主创新的投入，强化企业技术创新主体地位，鼓励发展产业技术创新联盟，加快省级科技成果转化示范基地建设，充分释放科技创新潜力。大力发展高新技术产业，实现全市高新技术企业突破100家，高新技术产业增加值占GDP比重不低于8%。积极推进“两化”融合，充分利用物联网、大数据、人工智能等新一代信息技术改造提升传统产业，推动生产组织、企业管理、商业运营模式创新。全面推进智慧城市建设，加强信息基础设施建设，编制城市地下空间规划，完善数字城管平台和城市地理信息中心。深入实施质量兴市战略，加强企业标准、计量和质量管理，加快市检测中心建设。鼓励企业研发创新、申请专利，让创新成果得到保护、创新权益得以实现。

优化金融支持。实施金融领先战略，大力推进市域金融工程。积极引进银行、证券、租赁、互联网金融等金融机构，打造咸宁金融港。组建国金公司、金融担保集团，设立大学生创业天使基金，用好红土成长基金、创投基金。鼓励银行创新金融产品，加大信贷投放力度。深化农村信用社改革，发展普惠式金融。启动社会征信体系建设。加快发展多层次资本市场，鼓励和引导本市企业在“新三板”及股权托管交易中心挂牌，组织开展企业股权融资，积极培育上市后备企业，力争实现企业上市零突破。推进与上海文化产权交易所合作，加快建设林业生态产业（文化）产权交易中心、低碳产业文化服务中心，打造中国文化生态金融融合区。

推进香城创业计划。建立公平开放透明的市场规则，实行统一的市场准入制度，鼓励各类市场主体平等进入负面清单之外领域。完善扶持创业的政策措施，全面推行“先照后证”登记制，对营业执照、组织机构代码证、税务登记证等实行一次申报、同步办理，减免注册登记费。抓好创业平台建设。大力发展混合所有制经济，加快发展民营经济，善待和支持小微企业，让更多人想创业、能创业、创大业。加大“招硕引博”力度，推进院士工作站建设，建立柔性引才机制，广揽各类人才。大力发展职业教育，抓好职业技能培训，使职业能力成为创新创造的“敲门砖”，使职业素养成为成功成才的“金钥匙”。

（五）做优做美绿色城乡

深度打造“香城泉都”城市品牌。以绿色崛起发展规划为引领，编制完善“香城泉都”总体规划及专项规划。围绕建设秀美、浪漫、和谐、生态的魅力城市，实施“香城泉都”品牌建设工程，谋划建设南外环高速，加快推进东环线、咸通高速马桥互通连接线、丹凤大桥、龙潭大桥、桂花桥、西环线滨水景观带、中心花坛区域综合改造等项目建设，确保如期实现第十届中国（武汉）园博会咸宁分园开园、天香温泉小镇开街、大型实景神话剧《嫦娥》开演的“三开”目标。建设茶博馆、李邕书画院、咸宁记忆馆、档案图书馆等项目，延续城市历史文脉。积极推进海绵城市建设，增强城市生态功能。加大棚户区改造、城中村改造、城乡结合部改造力度，推进市区一体城市综合管理，让秀美、干净、整洁成为城市常态。完善城市交通体系，落实公交优先发展战略，让市民安全出行、方便出行。巩固国家卫生城市创建成果，进一步抓好国家森林城市、国家环保模范城市、国家双拥模范城市争创工作，推动创建国家生态园林城市。继续办好国际温泉文化旅游节，提升城市美誉度和影响力。

积极推进新型城镇化，建设美丽乡村。认真落实全域新型城镇化总体规划，坚持一张蓝图绘到底、一张蓝图干到底。全力推动市域中心建设，以“四城四区”为支点，推动市区北拓东展西进，形成开放型、组团式发展格局。开展市县“多规合一”试点，推动县市城区与周边城镇协调互动、组合发展。突出抓好重点中心镇、特色镇建设，支持赵李桥、贺胜桥、潘家湾、麦市、白霓、九宫山等打造明星城镇。大力开展“四边三化”整治行动，加强农村道路、安全饮水、农田水利等基础设施建设，改善农村生产生活条件。结合开展“三万”活动，确保实现村村通客车。完善乡村发展规划，保护传统村落，保护乡情美景，积极创建“宜居村庄”，推进“一五一十”工程全覆盖，实现乡村与城镇、自然与人文各美其美、美美与共。

（六）坚持不懈推进生态文明建设

加大生态保护和环境治理力度。把生态文明建设放在更加突出地位，全力保护好蓝天白云、青山绿水。大力开展“绿满鄂南”行动，重点抓好“山上再造”、城乡绿化、林网建设三大生态工程，加快推进绿色示范乡村、省界门户绿化等生态示范项目建设，完成年度造林49.3万亩，确保实现三年绿色全覆盖。深化大气污染治理，强化机动车尾气整治，开展淘汰黄标车和老旧车工作，严格控制工地、道路扬尘及油烟污染。加快国家林业生态定位站、航空护林站等项目建设。完善饮用水源地保护规划，推进水源涵养、湿地保护和生态隔离带建设，建设一批应急备用水源项目，加大淦河、陆水等流域水污染防治力度，完成陆水分段监测站建设任务，确保饮用水源安全。建立单月环境会诊制度，开展环保“春雷”行动，加强环境监管，确保不发生重特大环境污染事故。

推进资源节约集约利用。严格落实节能减排责任制，淘汰落后产能，控制污染物排放，推进约谈问责、限期整改和区域限批常态化。进一步强化用地管理，实行最严格的耕地林地保护制度，打好闲置低效用地处置、GDP地耗考核、土地利用综合监管攻坚战。集约开发高岭土、石灰石等矿产资源，突出抓好非煤矿山整治，严禁违法开山采石，严厉打击河道非法采砂。加大地质灾害防治力度。积极推广绿色建筑、绿色照明、绿色交通和绿色消费，让绿色成为城市的主色调。

加强生态文明制度建设。积极开展水生态文明城市建设试点，推进创建国家生态保护和建设示范区。全面开展空气环境质量、PM2.5、PM10监测，推进环境信息公开。大力弘扬生态文化，广泛开展绿色城镇、绿色村庄、绿色企业、绿色学校、绿色社区、绿色家庭等创建活动，推动形成尊重自然、节约资源、爱护环境、保护生态的良好社会风尚。

（七）切实改善民生福祉

全力以赴保民生。越是经济下行，越是财力紧张，越要把保障和改善民生放在重要位置。实施更加积极的就业政策，坚持稳定和扩大就业并重，确保登记失业率控制在4.6%以内。全面实施社会保险全民参保登记计划，确保社会保险待遇按时足额发放。加强城乡低保动态管理。落实被征地居民参加养老保险。完善城乡养老服务体系，加快市老年人活动中心建设，推进社区居家养老服务工作，全面落实老年人优待政策。进一步完善临时救助、大病医疗救助制度。统筹推进保障性住房、移民安置建房和农村危房改造，确保困难群众有房住。以提前三年全面建成小康社会目标统领开发扶贫工作，加大幕阜山片区区域发展与扶贫攻坚力度，健全精准扶贫机制，确保实现减贫10万人。

与时俱进惠民生。优先发展教育事业，促进教育公平，让每个孩子都有人生出彩的机会。整体推进全市义务教育均衡发展，确保所有县（市、区）全部通过省级以上评估验收。加快薄弱学校改造，推进教育信息化建设，加大师资交流力度，以提高教学质量为核心，全面推进素质教育。支持高校转型发展，增强服务地方经济社会发展的能力。加快发展医疗卫生事业，做好疾病预防控制，强化药品安全监管、阳光配送，促进基本公共卫生服务均等化。加强出生人口性别比综合治理，严格控制政策外生育。积极创建国家公共文化服务体系示范区，大力弘扬社会主义核心价值观，广泛开展群众性文化活动，推动全民阅读，推进县、乡、村三级文化设施提档升级。支持文化艺术精品创作，加强地域文化遗产保护，打造咸宁文化品牌。大力发展全民健身、竞技体育和体育产业。繁荣和发展哲学社会科学。推进社区科普大学建设，提高全民科学素养。大力发展慈善公益事业，加强残疾人保障服务体系建设，加强未成年人保护工作，维护妇女儿童合法权益。认真做好民族宗教、外事侨务、对台事务、档案方志、对口支援等工作，推动各项社会事业迈上新台阶。

共建和谐利民生。加强和创新社会治理，有效预防和化解社会矛盾。全面推进依法治市，深化全民普法，进一步做好法律服务和法律援助工作。完善基层信访网络，构建民情“立交桥”，引导涉法涉诉信访问题在法治轨道上解决。切实抓好严重精神障碍人员、吸毒人员、刑释解教人员等三类特殊人群的服务管理，建立特殊人群帮扶基地，树立“咸宁帮扶”品牌。深入推进“平安咸宁”建设，加强社会治安综合治理，创新立体化社会治安防控体系，依法严密防范和打击网络诈骗、传销、涉毒、涉黑等违法犯罪活动，提高反恐防恐能力，确保社会和谐稳定。毫不放松抓好安全生产，坚决遏制重特大事故。重视做好防洪、抗旱、森林防火、气象和地震防灾减灾工作。加强信息安全和保密工作。加强国防动员和后备力量建设，做好人防工作，支持军队、武警部队建设，支持法院、检察院依法独立公正行使职权。更好地发挥工会、共青团、妇联等群团组织作用。

今年，市政府继续为民办好“十件实事”：（1）新增城镇就业４万人，实现社会保障卡持卡人数120万人；新建创业基地（孵化器）8个，扶持1万名大学生和市民创业，发放政府贴息小额担保贷款2.2亿元。（2）实施“放心餐饮”工程，打造“放心餐饮”示范店，确保餐饮服务食品安全。（3）建立农村特困居民大病医疗救助基金，为农村特困居民实施大病医疗救助。（4）实施饮水安全工程，加大南川水库水源地保护力度，确保王英水库引水工程顺利完成。（5）改善城区交通环境。完成肖桥大道建设，打通市温泉中学、实验小学东通道，缓解希望桥交通压力；建设鄂高地下通道，解决咸宁大道鄂高段安全通行问题；打通三江至太乙路通道，缓解一号桥交通压力。（6）发放农村居民健康卡208万张，改扩建10家乡镇卫生院、100家村卫生室；为幕阜山片区贫困适龄妇女免费进行“两癌”检查。（7）持续推进义务教育均衡发展。全面通过义务教育均衡发展评估验收；推进师资交流和信息化互联互通，让农村孩子享受到优质教育资源。（8）加强农村（社区）党员群众服务中心建设，新（改）建村级综合服务社100家。（9）丰富城乡文化生活，举办文化惠民演出活动1400场，完成408个行政村的农村智能广播网（村村响）工程建设。（10）为困难群众和农村居民无偿提供法律援助2000件以上。

三、以建设法治政府为核心，加强政府自身建设

全面推进依法行政。把依法行政贯穿政府工作始终，自觉养成依法办事的习惯，确保政府一切工作在法律框架内运行。坚持科学决策、民主决策、依法决策，健全重大行政决策规则，推行法律顾问制度，建立健全重大决策合法性审查制度、终身责任追究制度及责任倒查机制。加强政府智库建设。推进综合执法，加大安全生产、食品药品安全、公共卫生、环境监管等重点领域执法力度，严格规范执法行为。健全行政裁量权基准制度，提高执法效率和规范化水平。严格执行行政执法和刑事司法衔接制度。全面落实行政执法责任制，加强执法监督，建立执法全过程记录制度，推进执法信息化建设。

强化权力运行监督。坚决维护市委的领导，自觉接受人大及其常委会的法律监督、工作监督和政协的民主监督，主动接受新闻舆论和社会公众监督，广泛听取各方意见，认真做好人大代表议案、建议和政协委员提案办理工作。加强对政府内部权力的制约，对政府性资金使用、政府性工程建设、公共资源交易、国有资产监管等权力集中的部门和岗位实行分事行权、分岗设权、分级授权、定期轮岗，防止权力滥用。加大政务公开力度，重点推进财政预算、公共资源配置、重大项目建设、机构编制等领域的政府信息公开。整合公共资源交易中心，建立政府大数据平台，完善网上办事大厅和移动审批平台，建成永不下班的“线上政府”。支持审计、监察等部门依法独立行使监督权。强化行政负责人推进依法行政第一责任，建立完善纠错问责机制，强化对违纪违法行为的责任追究。

加强廉政建设，提高政府执行力。认真落实党风廉政建设主体责任，坚持“一岗双责”和“力度统一”，严格执行中央八项规定、省委六条意见和市委七项要求，始终做到心中有党、心中有民、心中有责、心中有戒。牢固树立“过紧日子”的思想，把有限的资金用足、用活、用在刀刃上，确保“三公”经费“零增长”。面对经济发展新常态，各级政府及其部门要自觉守纪律、讲规矩，加强上下联动、团结协作，确保政令畅通、执行有力。各级公务人员要振奋精神、用心谋事、主动干事、敢于担当作为，争当“两为”干部。大力推进学习型机关建设，学好党的理论及市场课程，完善知识结构，解决“本领恐慌”问题。完善政府目标责任体系，强化绩效考核，全面提高政府行政效能。巩固党的群众路线教育实践活动成果，积极回应社会关切，多为群众办实事，多做雪中送炭的工作，持之以恒抓作风、改作风，以狠抓落实的实际成效服务群众、取信于民。

各位代表！发展成就令人鼓舞，改革势头催人奋进。让我们更加紧密地团结在以习近平同志为总书记的党中央周围，在市委的领导下，凝聚全市人民的智慧和力量，解放思想，乘势而上，敢做敢为，善作善成，加快推进省级战略咸宁实施干在实处走在前列，满怀信心建设咸宁更加幸福美好的明天！
